# Molecular characteristics and advances in precision therapeutics for HR-positive and HER2-low breast cancer

**DOI:** 10.3389/fonc.2026.1857207

**Published:** 2026-07-17

**Authors:** Yu Xia, Jiawei Song, Haiyan Zhang, Shiwei Liu, Jing Luo

**Affiliations:** 1 Department of Breast Center, West China Second University Hospital, Sichuan University, Chengdu, China; 2 Key Laboratory of Birth Defects and Related Diseases of Women and Children, Sichuan University, Ministry of Education, Chengdu, China

**Keywords:** antibody-drug conjugate, endocrine therapy, HR-positive HER2-low expressing breast cancer, molecular characteristics, precision diagnosis and treatment

## Abstract

HR-positive HER2-low-expressing breast cancer is a unique subtype that has been refined from the traditional HER2 binary classification system in recent years, accounting for nearly 60% of the overall incidence of breast cancer. It has significant value for precise diagnosis and treatment research. This review systematically elaborates on the epidemiological characteristics, biological basis, diagnostic technology progress, and treatment strategy evolution of this subtype. HR-positive HER2-low-expressing breast cancer is characterized by the dominance of the estrogen receptor pathway and the core molecular feature of the intersection of HER2 low-expression signals. Its diagnosis requires a balance between the fundamental role of endocrine therapy and the precise screening of anti-HER2 targeted therapy. In terms of diagnosis, the initial screening by immunohistochemistry combined with fluorescence *in situ* hybridization has formed a standardized process. The integration of digital pathology, next-generation sequencing, and circulating tumor DNA in liquid biopsy technologies has effectively improved diagnostic accuracy and dynamic monitoring capabilities. In terms of treatment, endocrine therapy is the cornerstone, and the CDK4/6 inhibitor combination regimen is the standard for advanced first-line treatment. Antibody-drug conjugates, especially deruxtecan, have significantly improved patient prognosis and reshaped the treatment landscape. In summary, HR-positive HER2-low-expressing breast cancer has entered the era of individualized precise treatment guided by molecular typing. In the future, further exploration of new biomarkers, optimization of combination therapy strategies, and high-quality clinical research are needed to continuously improve patient survival outcomes.

## 1 Introduction

At present, breast cancer is the malignant tumor with the highest incidence among women worldwide. Its incidence has been increasing year by year and demonstrates a trend toward younger age of onset. The treatment of breast cancer primarily depends on tumor staging and molecular subtyping. Human epidermal growth factor receptor 2 (HER2) is a transmembrane tyrosine kinase (TK) receptor that forms active dimers with another member of the HER receptor family, epidermal growth factor receptor (EGFR) (also known as HER1). Subsequently, phosphorylation of tyrosine residues on the receptors leads to the activation of intracellular signaling pathways, thereby promoting cell growth and proliferation (1). In the 1980s, it was identified as being associated with poor prognosis in breast cancer (2), with positive expression observed in approximately 15% of breast cancer patients. The discovery of this target heralded the era of molecular targeted therapy for breast cancer, and HER2 status began to be reported clinically in a binary classification (i.e., HER2-positive/negative). Immunohistochemistry (IHC) and fluorescence *in situ* hybridization (FISH) are the principal methods for assessing HER2 status. According to the 2018 HER2 testing guidelines issued by the American Society of Clinical Oncology (ASCO) and the College of American Pathologists (CAP) (3), pathologists classify the expression status based on the intensity of HER2 staining (4)., Cases classified as IHC 0/1+ or IHC 2+ with no HER2 gene amplification detected by FISH are considered negative, whereas cases classified as IHC 3+ or IHC 2+ with HER2 gene amplification confirmed by FISH are considered positive. Subsequently, the continuous introduction of agents such as trastuzumab, pertuzumab, and tyrosine kinase inhibitors (e.g., neratinib) has significantly improved the prognosis of patients with HER2-positive breast cancer.

However, with the increasing precision of breast cancer diagnosis and treatment, studies have revealed that nearly 60% of breast cancers previously classified as HER2-negative actually meet the criteria for HER2-low expression (5), the categorization of HER2 status is evolving from the traditional binary “positive/negative” system toward a more refined classification that incorporates HER2-low expression. HER2-low expression is defined as IHC 1+ or IHC 2+ with negative FISH. The breakthrough findings of the DESTINY-Breast 04 trial (6) in 2022 confirmed the significant efficacy of trastuzumab deruxtecan (T-DXd) in patients with HER2-low breast cancer, thereby directly driving the establishment of the concept of HER2-low expression. Subsequently, a standardized definition was formally delineated at both international and national levels. This academic consensus is fundamentally grounded in evidence-based support from clinical trials such as DESTINY-Breast 04 and DESTINY-Breast 06 (7), as well as molecular characterization studies revealing the biological heterogeneity between HER2-low tumors and conventional HER2-negative tumors. Its clinical implication lies in facilitating the therapeutic shift for this patient population from chemotherapy to antibody–drug conjugate (ADC) therapy, while also prompting guidelines such as those from the ASCO and the CAP to mandate explicit reporting of HER2-low status in pathology reports (8).

As the exploration of the HER2-low expression field advances, studies have revealed that the majority of patients in this subgroup exhibit hormone receptor (HR)-positive status (65–83%) (1). HR-positive breast cancer is defined as breast carcinoma in which estrogen receptor (ER) and/or progesterone receptor (PR) immunohistochemical staining demonstrates positivity in ≥1% of tumor cell nuclei (9). HR-positive, HER2-low breast cancer, owing to the positivity of ER and/or PR, this subtype demonstrates a natural therapeutic advantage in response to endocrine therapy, which constitutes the cornerstone of early-stage management; however, it is also prone to primary or acquired endocrine resistance during long-term treatment, with a subset of patients exhibiting an elevated risk of increased tumor proliferative activity. Concurrently, the low expression of HER2 disrupts the conventional binary classification of HER2-positive versus HER2-negative disease, thereby providing a potential actionable target for novel anti-HER2 agents, such as ADCs. Consequently, therapeutic decision-making for this subtype must integrate both the foundational role of endocrine therapy and the precision-targeted approach offered by anti-HER2 strategies. This review focuses on the biological characteristics, diagnostic methodologies, and therapeutic strategies pertaining to this subtype, with the aim of informing both clinical practice and future research directions.

## 2 Epidemiological characteristics of HR-positive HER2-low expressing breast cancer

Schettini et al. (10)analyzed data from 3,689 patients with HER2-negative breast cancer and found that HER2-low breast cancer accounted for 59.7% of all breast cancer cases. Among HR-positive patients, the proportion of HER2-low expression (65.4%) was significantly higher than that in triple-negative breast cancer (36.6%). Moreover, compared with patients with HER2-negative tumors, HER2-low breast cancer was more frequently observed in older patients and was associated with more extensive axillary lymph node involvement. In addition, data from the U.S. Surveillance, Epidemiology, and End Results (SEER) (11)program indicate that the incidence of the ER(+)/HER2(–) subtype (encompassing HER2-low) is highest among White women (75.5%), followed by Asian/Pacific Islander women (71.1%), and lowest among Black women (60.2%).

## 3 Biological characteristics

**Figure 1:**
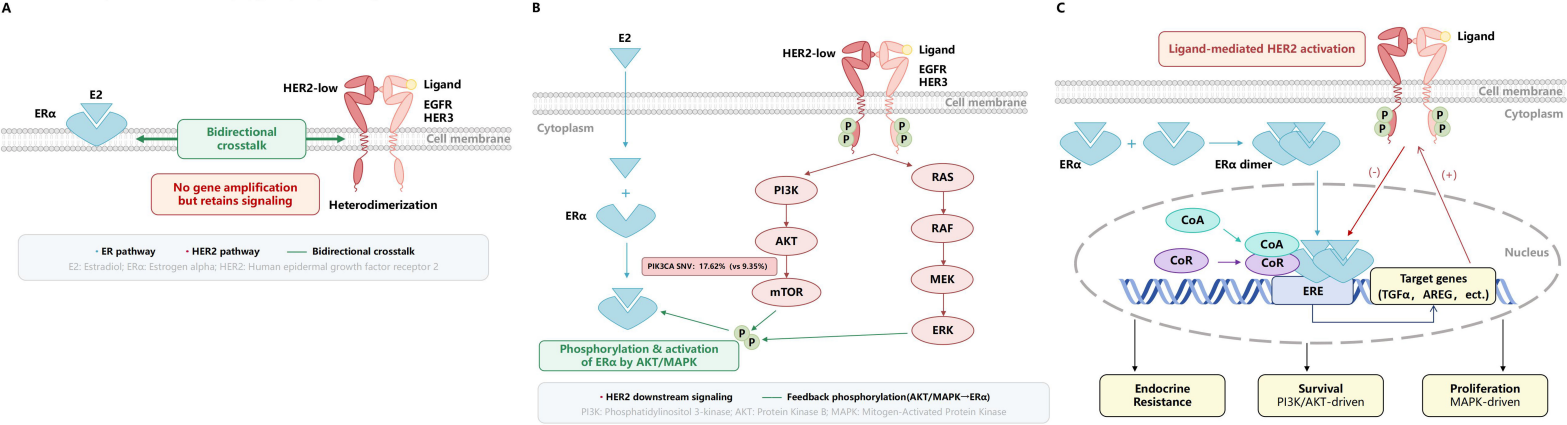
**(A)** At the membrane receptor level: estradiol (E2) binds and activates ERα; HER2 is expressed at low levels (IHC 1+ or IHC 2+/FISH−), retaining downstream signaling capability by forming heterodimers with EGFR or HER3. A green bidirectional arrow indicates reciprocal cross-regulation between ERα and HER2. **(B)** Intracellular signaling and cross-phosphorylation: HER2-low expression activates the PI3K/AKT pathway (driving cell survival) and the RAS/RAF/MEK/ERK pathway (driving cell proliferation). Simultaneously, AKT and MAPK signals reciprocally phosphorylate and activate ERα via the green dashed line. The figure highlights the core molecular feature of this subtype—significantly enriched PIK3CA single-nucleotide variant frequency (17.62% vs. 9.35%). **(C)** Nuclear transcription, feedback loops, and biological outcomes: After dimerization, ERα enters the nucleus, binds to estrogen response elements (ERE), and recruits coactivators (CoA) or corepressors (CoR), initiating the transcription of target genes (e.g., TGFα, AREG). These genes then enhance HER2 signaling through ligand-mediated feedback (green dashed feedback loop), while HER2 signaling can also suppress ER-driven transcription. Ultimately, this cross-regulation collectively drives cell proliferation, survival, and resistance to endocrine therapy. At the bottom, key clinicopathological features of this subtype are summarized.

The core molecular feature of HR-positive, HER2-low breast cancer is characterized by the coexistence of ER/PR positivity and HER2-low expression. Studies (12) have shown that compared with HER2-negative breast cancer, HER2-low breast cancer is associated with better overall survival, more favorable prognosis, lower histologic grade, and a lower incidence of lymph node metastasis. However, after adjusting for HR status, the prognostic and clinicopathologic significance of HER2-low expression was no longer evident. These findings indicate that the improved prognosis of HER2-low tumors is closely linked to HR status, and that HER2-low tumors do not represent a distinct biological or prognostic subtype of breast cancer. Furthermore, the study (12) also demonstrated that over 90% of HER2-low breast cancers are HR-positive, this indirectly reflecting a close molecular association between low HER2 expression status and the luminal phenotype. As a transmembrane tyrosine kinase receptor, HER2 can form heterodimers with EGFR or ERBB3 even in the absence of gene amplification, and low-level HER2 expression remains capable of activating the downstream PI3K-AKT cell survival pathway and the RAS-MAPK proliferation/migration pathway (13) (See **Figure 1**), while also interacting synergistically with the ER signaling pathway to jointly promote the acquisition of a malignant tumor cell phenotype (14). Such crosstalk among these signaling pathways may constitute a key mechanism underlying primary or acquired resistance to endocrine therapy in this breast cancer subtype; notably, when concomitant with aberrant activation of the PI3K pathway, the risk of endocrine therapy resistance in affected patients is substantially elevated.

In addition to crosstalk-mediated drug resistance, HR-positive, HER2-low breast cancer exhibits a distinct genomic mutational spectrum, characterized by a significantly elevated frequency of single nucleotide variants (SNVs) in PIK3CA(17.62% vs. 9.35%) (15). PIK3CA mutations confer enhanced tumor cell survival and resistance to endocrine therapy through constitutive activation of the PI3K-AKT pathway, thereby serving as an important adverse prognostic marker and therapeutic target in this entity (16, 17). Furthermore, classic mutated genes of luminal breast cancer, including ESR1, GATA3, and CDH1, are also commonly observed in this subtype. Notably, ESR1 mutations have been implicated in endocrine therapy resistance and late-stage metastasis (18). In breast tissue, GATA3 is produced by luminal progenitor cells and functions as a core transcriptional regulator governing luminal epithelial differentiation. It has been demonstrated to exert tumor-suppressive effects, suggesting that GATA3 mutations may be closely associated with the initiation and progression of luminal breast cancer (19).

## 4 Diagnostic techniques and standardization for HR-positive HER2-low expressing breast cancer

Currently, in the diagnosis of HR-positive, HER2-low breast cancer, a unified international consensus has been established regarding the criteria for defining HR positivity. In contrast, the diagnosis of HER2-low expression remains fraught with limitations, including discrepancies in the clinical application of IHC scoring thresholds, insufficient inter-platform concordance of test results, and the complexity of requiring multiple orthogonal techniques for confirmation in borderline cases. The existing diagnostic paradigm centers on a core workflow of initial IHC screening followed by FISH confirmation, supplemented by NGS and liquid biopsy technologies for the dynamic assessment of therapeutic response.

### 4.1 Standardization of IHC testing

As the preferred method for initial HER2 status screening, IHC encompasses three core components in its standardized workflow: antibody selection, staining procedure, and interpretation of results. Regarding antibody selection, the use of specific antibodies approved by the U.S. Food and Drug Administration (FDA) or validated in accordance with ASCO/CAP guidelines is recommended, such as the 4B5 monoclonal antibody, which precisely recognizes the extracellular domain of the HER2 protein and reduces false-positive results attributable to cross-reactivity (20). Quality control of the staining procedure requires strict adherence to standardized operating procedures (SOPs), including tissue fixation time (10% neutral buffered formalin fixation for 6–72 hours), antigen retrieval method (high-temperature/high-pressure retrieval preferred over microwave retrieval), and calibration of the chromogenic detection system, along with regular use of positive and negative controls to verify assay validity (21).

Standardization of result interpretation is key to reducing interobserver variability. To further minimize interpretation discrepancies, digital pathology–assisted interpretation systems have been progressively implemented in clinical practice. These systems employ artificial intelligence algorithms for quantitative analysis of stained images, enabling accurate calculation of the proportion of positive cells and staining intensity. Their diagnostic concordance (Kappa value 0.85–0.92) is significantly higher than that of manual interpretation (Kappa value 0.68–0.75) (22). In addition, participation in external quality assessment (EQA) programs and targeted training for pathologists constitute essential measures to enhance interpretation accuracy.

### 4.2 Application scenarios and optimization of FISH testing

In HR-positive, HER2-low breast cancer, FISH analysis effectively distinguishes true low expression (non-amplified) from potentially amplified cases within the IHC 2+ subgroup, thereby preventing erroneous therapeutic decision-making regarding targeted therapy. Nevertheless, conventional FISH testing has certain limitations, including stringent requirements for specimen quality (23), a relatively prolonged turnaround time (24), and inherent challenges in accurately assessing HER2 expression heterogeneity arising from intratumoral variability (25).

In response to the aforementioned issues, a series of improved techniques have emerged in recent years: ① dual-color silver-enhanced *in situ* hybridization (SISH), which amplifies signals via silver deposition to enhance detection sensitivity for low-copy-number genes, with results observable under standard light microscopy, thereby facilitating wider adoption in primary care hospitals (26); ② single-molecule RNA *in situ* hybridization (RNAscope), which enables direct detection of HER2 mRNA levels and circumvents interference from post-translational modifications on protein expression, thus providing significant supplementary value for cases with equivocal IHC results (27); ③FISH analysis of circulating tumor cells (CTCs), which allows HER2 gene assessment in CTCs captured from peripheral blood and offers a non-invasive diagnostic avenue for patients from whom tissue samples cannot be obtained (28). Optimization of these technological applications has further broadened the scope of FISH-based detection in the diagnosis of luminal-type breast cancer with low HER2 expression.

### 4.3 Clinical applications of next-generation sequencing

NGS technology enables precise assessment of HER2 status, refinement of molecular subtyping, and concurrent detection of resistance-associated genes through approaches such as whole-exome sequencing (WES) and targeted capture sequencing. Regarding HER2 status evaluation, NGS allows direct detection of HER2 gene copy number variations (CNVs), point mutations, and fusion events. In cases where IHC and FISH yield discordant or inconclusive results, NGS can provide more comprehensive molecular evidence (29). For instance, NGS may identify low-level HER2 gene amplification or functional mutations in some IHC 1+ cases, thereby revealing potential therapeutic targets for anti-HER2 therapy.

In terms of refining molecular subtyping, NGS can further stratify HR-positive breast cancer into luminal A, luminal B, and luminal androgen receptor (LAR) subtypes based on multigene expression profiles (30), Among these, HR-positive, HER2-low breast cancer is predominantly enriched in the luminal B subtype and is frequently associated with mutations in genes such as phosphatidylinositol-4,5-bisphosphate 3-kinase catalytic subunit alpha (PIK3CA) and Estrogen Receptor 1 (ESR1) (31, 32). These molecular features not only inform prognosis but also guide the development of individualized treatment strategies. Furthermore, NGS enables simultaneous detection of gene alterations linked to resistance to anti-HER2 therapy and endocrine therapy (e.g., PIK3CA, AKT1, ESR1, TP53), thereby providing crucial evidence for investigating resistance mechanisms and adjusting therapeutic approaches (31). Currently, NGS is progressively transitioning from a research tool to a clinical application and has emerged as an essential instrument for the precise diagnosis and treatment guidance of HR-positive, HER2-low breast cancer.

### 4.4 Research progress and application prospects of liquid biopsy technology

Liquid biopsy technology, targeting circulating tumor DNA (ctDNA), CTCs, and other analytes, offers the advantages of noninvasiveness, real-time monitoring, and repeatability, demonstrating significant potential in the dynamic surveillance of HR-positive, HER2-low breast cancer. ctDNA analysis enables real-time reflection of dynamic alterations in tumor HER2 status through the detection of tumor-specific genetic mutations or gene copy number variations in peripheral blood. Studies have shown that during neoadjuvant therapy, changes in HER2 gene copy number in ctDNA are closely correlated with treatment response; patients who respond favorably exhibit a marked decline in ctDNA HER2 levels, whereas those with drug resistance maintain persistently elevated or rising levels (33). Moreover, ctDNA testing can detect molecular signals of tumor recurrence months or even years before radiological evidence of relapse, thereby providing a critical temporal window for early intervention (34).

As another crucial modality of liquid biopsy, CTC detection enables the analysis of HER2 protein and gene status by capturing tumor cells from peripheral blood. Studies (35) have demonstrated that the HER2 status determined via CTC detection exhibits favorable concordance with that of the primary tumor, and that alterations in CTC count and HER2 status may serve as independent indicators for assessing therapeutic response and predicting prognosis. Nevertheless, liquid biopsy techniques currently face persistent challenges, including limited detection sensitivity (particularly in early-stage patients) (36), relatively high testing costs (37), and the absence of standardized procedures (38). With ongoing technological advancements—such as the integration of digital PCR and single-cell sequencing—liquid biopsy is poised to become a routine approach for the diagnosis and long-term surveillance of HR-positive, HER2-low breast cancer.

**Figure 2:**
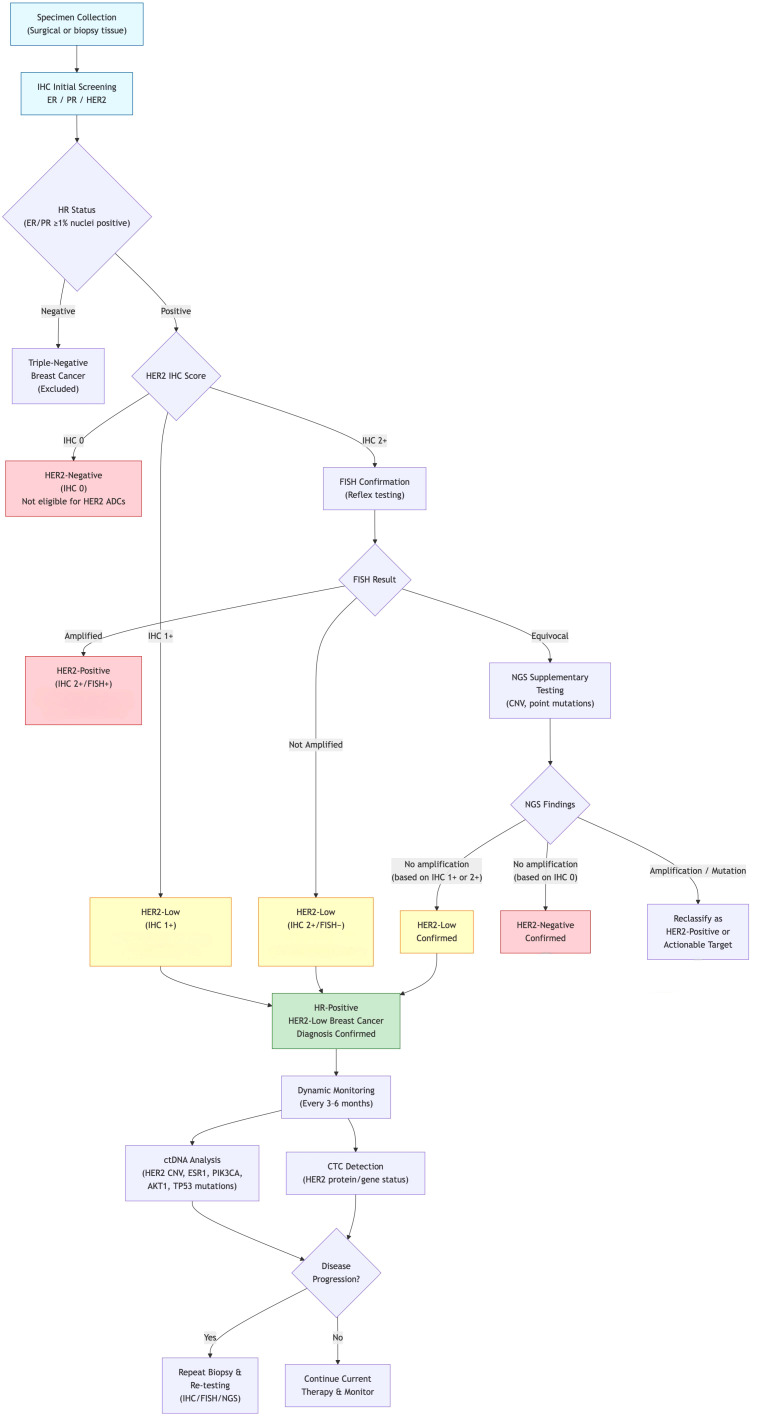
Standardized diagnostic procedure for HR-positive HER2-low expressing breast cancer.

Therefore, in accordance with the current ASCO/CAP guidelines, breast cancer diagnosis and treatment guidelines, and clinical practice, the standardized diagnostic workflow for HR-positive, HER2-low breast cancer should regulate the following key points(See **Figure 2**): For specimen collection, fresh surgical or biopsy tissue should be prioritized. The testing procedure consists of three steps: First, initial screening for HER2, ER, and PR status by IHC—cases with IHC 0/1+ and HR positivity are considered candidates, IHC 2+ cases require further FISH testing, and IHC 3+ cases are excluded from HER2-low designation. Second, FISH confirmation is performed for IHC 2+ cases—negative FISH results with HR positivity confirm the diagnosis, positive FISH results classify the case as HER2-positive, and equivocal results necessitate supplementary NGS. Third, during treatment, dynamic monitoring of HER2 status and resistance-associated gene alterations via ctDNA should be conducted every 3–6 months, with repeat testing upon disease progression. Concurrently, regular physician education on guideline updates, training in IHC interpretation for pathologists, and instruction in novel technical procedures should be implemented to ensure diagnostic accuracy and consistency.

## 5 Targeted treatment strategies for HR-positive HER2-low expressing breast cancer

Endocrine therapy and targeted ADC drugs for HER2 low-expression are the core treatment methods. Specifically, endocrine therapy leverages the subtype’s dependence on estrogen signaling, functioning as a fundamental regimen throughout both early- and late-stage disease management. Meanwhile, ADCs overcome the limited efficacy of conventional anti-HER2 agents in the low-expression population, offering a novel targeted option for patients who develop resistance to endocrine therapy or experience disease progression. The combined or sequential use of these approaches has driven a paradigm shift in managing this subtype—from endocrine monotherapy toward molecular marker-guided, individualized precision treatment.

### 5.1 Endocrine targeted therapy

Currently, stratified treatment based on menopausal status, disease stage, and risk of recurrence constitutes the core principle of endocrine therapy. For premenopausal patients with early-stage disease, selective estrogen receptor modulators (SERMs) such as tamoxifen combined with ovarian function suppression (OFS) are recommended for those at low risk, whereas tamoxifen alone provides insufficient long-term control of distant recurrence risk in a subset of patients at intermediate to high risk. Therefore, selection of endocrine therapy drugs and whether to combine with OFS has become a key variable influencing long-term outcomes. This question has been addressed by two large-scale, multicenter trials—SOFT and TEXT (39)—which demonstrated that OFS combined with endocrine therapy reduces the risk of recurrence in premenopausal patients with HR-positive early breast cancer. Among the regimens studied, exemestane plus OFS exhibited the most definitive long-term benefit. Subgroup analyses revealed that this benefit was predominantly observed in clinically high-risk populations, including patients requiring (neo)adjuvant chemotherapy, premenopausal women younger than 35 years of age, and those with grade 3 histology. In postmenopausal patients, aromatase inhibitors (AIs), including letrozole, anastrozole, and exemestane, are the preferred agents, with a standard treatment duration of five years; for patients at high risk of recurrence, extended AI therapy for an additional five years is warranted. Regardless of menopausal status, adjuvant intensification therapy is crucial for patients with early-stage intermediate to high-risk disease. The positive results of the monarchE trial (40) and the NATALEE trial (41) signify that the therapeutic strategy for HR-positive breast cancer has progressed from merely intensifying endocrine suppression toward precision intervention targeting tumor proliferative pathways. The monarchE trial was designed to investigate whether the addition of the cyclin-dependent kinase 4/6 (CDK4/6) inhibitor abemaciclib for two years to standard endocrine therapy could improve outcomes in patients with HR-positive, HER2-negative early high-risk breast cancer. The results demonstrated that abemaciclib plus endocrine therapy reduced the risk of death by 15.8%. Similarly, the NATALEE trial indicated that adjuvant therapy with ribociclib plus a nonsteroidal AI reduced the 5-year risk of invasive disease-free survival (iDFS) events by 28.4%, along with a 29.1% reduction in the risk of distant metastasis and a 30.1% reduction in the risk of distant recurrence. Furthermore, the DAWNA A study, led by Chinese investigators and presented at the 2025 ASCO Annual Meeting, has confirmed that the domestically developed CDK4/6 inhibitor dalpiciclib significantly improves iDFS and reduces the risk of invasive recurrence in patients with HR-positive, HER2-negative early breast cancer. The complete data from this study have not yet been formally published in full.

Endocrine therapy for early-stage patients is largely similar across regimens, whereas first-line endocrine therapy for advanced-stage patients is determined not only by menopausal status but also by prior treatment history. For postmenopausal patients, treatment options include endocrine therapy combined with targeted pathway inhibitors, such as inhibitors of CDK4/6, mammalian target of rapamycin (mTOR), or PIK3CA (42). The preferred first-line endocrine agents include AIs or selective estrogen receptor degraders (SERDs) such as fulvestrant. For premenopausal patients, OFS must be added to these regimens. Among these combinations, CDK4/6 inhibitors combined with endocrine therapy have become the standard first-line treatment for advanced HR-positive, HER2-low expressing breast cancer. The PALOMA-4 (43) study demonstrated that palbociclib combined with letrozole in advanced postmenopausal patients extended median progression-free survival (PFS) from 13.9 months to 21.5 months, with a consistent benefit trend observed in the luminal subtype subgroup compared to the overall population. The MONALEESA-2 (44) study of ribociclib plus an AI and the MONARCH-3 (45) study of abemaciclib plus an AI have similarly confirmed PFS benefits, with no significant differences in safety profiles among the three agents. The primary adverse reactions include neutropenia and fatigue, which are generally manageable through dose adjustments. However, endocrine resistance frequently develops in advanced-stage patients. The core mechanisms of resistance include ESR1 mutations, activation of the PI3K/AKT/mTOR pathway, aberrations in the CDK4/6 pathway, and reactivation of HER2 signaling (46).Notably, the incidence of ESR1 mutations in patients resistant to AIs reaches 36%, representing a primary cause of AI failure due to ligand-binding domain alterations (47). Consequently, targeted therapeutic strategies have been established to address specific resistance mechanisms: For patients with ESR1 mutations, fulvestrant or novel SERDs (e.g., elacestrant, giredestrant) are preferred, as these agents function independently of the ER ligand-binding domain. Certain novel agents, such as the PROTAC drug ARV-471, further enhance efficacy by promoting ER degradation (48). For patients with aberrations in the PIK3CA pathway, PI3Kα inhibitors combined with fulvestrant are indicated, The SOLAR-1 study (17) demonstrated that in advanced AI-resistant patients with PIK3CA mutations, alpelisib combined with fulvestrant extended median PFS from 5.7 months to 11.0 months, with an objective response rate (ORR) of 35.7%. Similarly, the INAVO120 study (49) indicated that in patients with PIK3CA-mutated, HR-positive locally advanced or metastatic breast cancer, inavolisib combined with palbociclib and fulvestrant improved PFS by 7.7 months compared to the control group. For patients with mTOR pathway activation, everolimus combined with an AI is recommended. The BOLERO-2 study (50) showed that compared to exemestane monotherapy, this combination extended median PFS from 3.2 months to 7.8 months and increased the ORR to 12.6%. However, this regimen is associated with a higher incidence of adverse events such as stomatitis and hyperglycemia, necessitating enhanced monitoring and timely intervention during treatment. Therefore, following progression on a CDK4/6 inhibitor or AI, treatment strategies may involve switching endocrine partners combined with alternative targeted agents, transitioning to chemotherapy, or initiating anti-HER2 therapy.

### 5.2 Drugs targeting HER2-low expression

The application of traditional anti-HER2 monoclonal antibodies, such as trastuzumab and pertuzumab, in the HER2-low population remains highly controversial. Multiple clinical studies have demonstrated that the objective response rate (ORR) of these agents—whether administered as monotherapy or in combination with chemotherapy—is generally below 15%, and they fail to significantly improve patient survival (51). Consequently, they are not recommended as a standard therapeutic regimen. In contrast, antibody–drug conjugates (ADCs) have broken through this impasse by virtue of their unique mechanism of “targeted delivery of cytotoxic agents,” yielding remarkable clinical benefits for patients. An ADC consists of an antibody, a cytotoxic payload, and a linker. Its principal mechanisms for tumor cell eradication include: specific recognition and binding of the antibody moiety to target antigens on the tumor cell surface, induction of tumor cell apoptosis following receptor-mediated endocytosis, and elimination of target antigen-negative tumor cells via the bystander effect (52). Furthermore, novel ADCs can achieve innovative functions such as immune activation and photo-controlled cytotoxicity through the incorporation of immunomodulators or photosensitive dyes, thereby establishing a multi-pathway synergistic anti-tumor framework characterized by “targeted delivery—intracellular killing—immune synergy—innovative potentiation.”

#### 5.2.1 Trastuzumab deruxtecan

Trastuzumab deruxtecan (T-DXd) is an antibody-drug conjugate comprised of a humanized anti-HER2 monoclonal antibody linked to a topoisomerase I inhibitor payload via a tetrapeptide-based cleavable linker. It effectively targets tumor cells with low HER2 expression and delivers its potent cytotoxic payload (with a high drug-to-antibody ratio of approximately 8:1) to neighboring tumor cells via a bystander effect, irrespective of their HER2 expression status (6). The considerable attention garnered by T-DXd stems primarily from the long-term follow-up data of the DESTINY-Breast (DB) series of clinical trials, which have demonstrated exceptional outcomes. This evidence has not only propelled the clinical application of T-DXd from advanced breast cancer towards earlier treatment stages but has also expanded the benefiting population from traditional HER2-positive patients to include those with HER2-low and even HER2-ultralow expression, thereby successfully breaking therapeutic boundaries in both disease staging and molecular subtyping of breast cancer. The DESTINY-Breast 04 trial (6), as the first pivotal study targeting unresectable or metastatic HER2-low breast cancer, was designed to evaluate the efficacy and safety of T-DXd compared to physician’s choice of standard chemotherapy in patients who had received one or two prior lines of chemotherapy in the metastatic setting. The results demonstrated superior median progression-free survival (PFS) and median overall survival (OS) in the T-DXd arm compared to the chemotherapy arm. Subgroup analysis further revealed a more pronounced benefit in patients with hormone receptor (HR)-positive breast cancer. These findings established T-DXd as the standard of care following chemotherapy for HER2-low metastatic breast cancer, encompassing both HR-positive and HR-negative populations. The DESTINY-Breast 06 trial (7) aimed to evaluate the efficacy of T-DXd in patients with HR-positive, HER2-low or HER2-ultralow metastatic breast cancer who had progressed on endocrine therapy but had not received prior chemotherapy in the metastatic setting. The results indicated that, compared to physician’s choice of chemotherapy, T-DXd significantly improved median PFS: in the HER2-low subgroup, median PFS was 13.2 months, an improvement of 5.1 months over chemotherapy. A similar advantage was observed in the HER2-ultralow subgroup. These findings address the therapeutic gap for HR-positive, endocrine-resistant patients who have not yet received chemotherapy, positioning T-DXd as a targeted therapy option before chemotherapy. Furthermore, this extends the benefit of anti-HER2 therapy to the HER2-ultralow population, further broadening the scope of precision medicine.

Beyond the HER2-low and HER2-ultralow populations, the performance of T-DXd in HER2-positive advanced breast cancer has also been striking. The DESTINY-Breast 03 study (53) compared the efficacy and safety of T-DXd versus ado-trastuzumab emtansine (T-DM1) in the second-line treatment of HER2-positive metastatic breast cancer. The results showed a significant improvement with T-DXd in both median PFS (28.8 months vs. 6.8 months) and median OS (52.6 months vs. 42.7 months). Consistent benefits with T-DXd were observed regardless of hormone receptor status, prior pertuzumab treatment, or the presence of visceral or brain metastases. This established T-DXd as the gold standard for second-line treatment of HER2-positive metastatic breast cancer, while also laying the groundwork for exploring its role in the first-line setting. Based on the significant success of T-DXd in the second-line setting, the DESTINY-Breast 09 study (54) challenged the standard first-line regimen of taxane plus trastuzumab and pertuzumab (THP) for HER2-positive advanced breast cancer. The study randomized patients into three arms: T-DXd monotherapy, T-DXd plus pertuzumab (P), and the standard-of-care THP regimen. An interim analysis (data cutoff: February 26, 2025) demonstrated that the T-DXd plus pertuzumab arm achieved a median PFS of 40.7 months, a significant prolongation of 13.8 months compared to the 26.9 months observed in the THP arm (hazard ratio for disease progression or death, 0.56). The objective response rate (ORR) was 85.1% in the T-DXd+P arm (vs. 78.6% in the THP arm), with a notable complete response (CR) rate of 15.1% (vs. 8.5% in the THP arm). Based on these results, the T-DXd plus pertuzumab regimen has been added as a Category 2A recommendation for first-line treatment of HER2-positive advanced breast cancer in the 2026.V1 NCCN Breast Cancer Guidelines and has received U.S. FDA approval. Another multicenter, open-label phase II clinical trial, the DAISY study (55), focused on the efficacy and safety of T-DXd in metastatic breast cancer patients across varying levels of HER2 expression. The study enrolled 186 patients with metastatic breast cancer and stratified them into three cohorts based on baseline biopsy HER2 status (HER2-positive, HER2-low, and HER2-null). All patients received the same T-DXd treatment regimen. After a median follow-up of 15.6 months, the efficacy data across the three cohorts clearly demonstrated an increasing trend in benefit corresponding with higher HER2 expression levels. This exploratory trial further validates the broad-spectrum activity of T-DXd across the HER2 expression spectrum. From improving long-term survival in advanced disease to raising hopes for cure in early-stage disease, and from redefining molecular subtypes to facilitating the clinical translation of biomarkers, the DESTINY-Breast series of trials serves as a paradigm for targeted cancer therapy and establishes a robust foundation for the future development and application of other antibody-drug conjugates.

#### 5.2.2 Other ADC agents

Disitamab vedotin (RC48), an anti-HER2 antibody–drug conjugate independently developed in China, is composed of a humanized anti-HER2 monoclonal antibody, a cleavable linker, and the microtubule inhibitor monomethyl auristatin E (MMAE), with an average drug-to-antibody ratio (DAR) of 4:1. It exhibits specific binding capability to HER2-low-expressing antigens. A phase I clinical study (56) evaluated the efficacy and safety of RC48 in heavily pretreated patients with HER2-low advanced breast cancer. The results showed an objective response rate (ORR) of 33.3% and a median progression-free survival (PFS) of 5.1 months, demonstrating unequivocal antitumor activity in patients whose disease had progressed after multiple lines of chemotherapy.

SYD985 consists of trastuzumab conjugated to the DNA-alkylating agent duocarmycin via a short polyethylene glycol linker, combining targeted delivery with potent cytotoxicity. A phase I cohort study (57) enrolled heavily pretreated patients with metastatic breast cancer, including both HER2-positive and HER2-low subgroups. In the SYD985-treated arm, the ORR was 33% and the median PFS reached 9.4 months. Further analysis of the HER2-low subgroup revealed an ORR of 27% in hormone receptor (HR)-positive patients and 40% in patients with triple-negative breast cancer. Although the study is limited by a relatively small sample size and the enrollment of predominantly heavily pretreated cases, the preliminary data nonetheless confirm that SYD985 possesses appreciable antitumor activity in HER2-low breast cancer, with a manageable safety profile.

Beyond conventional ADC agents targeting HER2, studies have demonstrated (58) that Trop-2 is broadly overexpressed in breast cancer, particularly in HR-positive disease, thus representing a key therapeutic target in this population. Sacituzumab govitecan (SG) is an ADC comprising the humanized anti-Trop-2 monoclonal antibody hRS7 conjugated to the topoisomerase I inhibitor SN-38 via the pH-sensitive linker CL2A. The TROPICS-02 trial (59) focused on the efficacy and safety of SG in heavily pretreated patients with HR+/HER2- metastatic breast cancer (including HER2-low disease). After a median follow-up of 12.75 months, the SG arm demonstrated superior median overall survival (OS) (14.5 months vs. 11.2 months) and median PFS (5.5 months vs. 4 months) compared with the chemotherapy arm. Subgroup analysis further revealed a median OS of 15.4 months in the HER2-low cohort treated with SG. As the first Trop-2–directed ADC to demonstrate a significant OS benefit in HR+/HER2- breast cancer inclusive of HER2-low disease, SG provides a new standard-of-care option for a refractory population that has progressed after multiple lines of therapy. Another Trop-2–targeted ADC, datopotamab deruxtecan (Dato-DXd), is composed of the humanized monoclonal antibody MAAP-9001a conjugated to the topoisomerase I inhibitor DXd via a GGFG tetrapeptide linker. The phase III TROPION-Breast01 study (60) enrolled patients with unresectable or metastatic HR-positive, HER2-low or HER2-negative breast cancer who had previously received endocrine therapy and one to two lines of systemic chemotherapy. The trial aimed to evaluate the efficacy and safety of Dato-DXd compared with investigator’s choice of chemotherapy. The results indicated that Dato-DXd conferred a statistically significant and clinically meaningful improvement in PFS relative to the control arm, along with a favorable and manageable safety profile.

#### 5.2.3 Other targeted agents

In addition to antibody–drug conjugates (ADCs), the development of bispecific antibodies and small-molecule tyrosine kinase inhibitors (TKIs) offers new avenues of exploration for hormone receptor (HR)-positive, HER2-low breast cancer. The bispecific antibody ZW25 (zanidatamab) simultaneously binds two distinct extracellular domains of the HER2 protein. In its phase I/II study (61), ZW25 combined with chemotherapy achieved an objective response rate (ORR) of 90.9% in patients with advanced HER2-positive breast cancer, with favorable tolerability and no significant additional toxicity. These data support further evaluation of ZW25 in both HER2-positive and HER2-low populations. Investigation of small-molecule TKIs remains at an early stage: a small-sample study in the HER2-low setting demonstrated promising efficacy of pyrotinib plus chemotherapy in patients with HER2-low expression (62); however, confirmatory phase III evidence is lacking. Future research should focus on combining highly selective HER2 TKIs with endocrine therapy to explore potential synergistic effects.

## 6 Challenges in HR-positive, HER2-low breast cancer

### 6.1 Complex heterogeneity in target expression and pathway regulation

The heterogeneity of HR-positive, HER2-low breast cancer is characterized by spatial and temporal variability as well as intricate pathway crosstalk. Within the same patient, HER2 expression determined by immunohistochemistry (IHC) may fluctuate between 1+ and 3+ across primary tumors and metastatic lesions, and among different metastatic sites, while also evolving dynamically over the course of treatment. Some lesions may convert from HER2-low to HER2-negative following therapy, whereas initially HER2-negative lesions may upregulate HER2 expression to low levels after endocrine treatment. Mechanistically, substantial crosstalk exists between the HR and HER2 signaling pathways: activation of the estrogen receptor pathway can modulate HER2 membrane expression, and conversely, HER2-low status may reciprocally influence the transcriptional activity of the HR pathway (63). Furthermore, stress stimuli within the tumor microenvironment can concurrently alter the expression of both targets via the NF-κB pathway (64). Unlike the HER2-overexpressing subtype, which is driven by stable genomic aberrations, target expression in this subtype lacks consistent genomic alterations as a foundation. This dynamic heterogeneity complicates the sustained applicability of therapeutic strategies and underscores the urgent need for biomarkers capable of dynamically monitoring dual-target expression and pathway activity to provide precise guidance for treatment adjustment.

### 6.2 Dual challenges in pathological detection and subtype interpretation

Currently, no domestic or international guidelines have established dedicated HER2 testing and interpretation criteria for this subtype. HR positivity can alter tumor cell morphology and antigen expression, substantially reducing interobserver agreement among pathologists regarding the intensity of membrane staining and the proportion of positive tumor cells. Moreover, distinguishing HER2 ultralow expression (faint staining in <10% of tumor cells) from HER2 1+ is particularly challenging in HR-positive tissues. Although the clinical significance of such ultralow-expressing lesions remains unclear, they are inherently difficult to identify with precision (65). Additionally, preanalytical tissue processing and variations in sensitivity among different IHC antibody brands can markedly affect the detection of HER2-low expression. HER2-low status may also confound the interpretation of HR positivity, leading to false-positive or weak-positive results. This mutual interference in dual-target detection further compromises the accuracy of pathological subtyping (66). There is an urgent clinical need to develop standardized HER2 testing workflows and dedicated interpretation guidelines, while reinforcing quality control throughout the entire testing process.

### 6.3 Clinical dilemmas in therapeutic strategies and drug combinations

The treatment of HR-positive, HER2-low breast cancer presents clinical dilemmas in drug selection, combination strategies, and efficacy evaluation, and the suitability of existing treatment paradigms requires further validation. Endocrine therapy serves as the cornerstone of management for this subtype; however, long-term exposure frequently leads to resistance. Following the development of resistance, the optimal timing and regimen for combination therapy with antibody–drug conjugates (ADCs) remain unstandardized. Premature combination may escalate toxicity, whereas delayed intervention risks missing the optimal therapeutic window. Currently, studies of approved HER2-targeted ADCs predominantly focus on later-line treatment. There is a paucity of large-scale, evidence-based medical data regarding the efficacy of combining ADCs with endocrine agents and CDK4/6 inhibitors in the first-line setting. Furthermore, the potential for overlapping toxicities associated with such combinations warrants urgent evaluation, as this may compromise patient tolerability. Simultaneously, conventional RECIST criteria often fail to accurately delineate the tumor regression patterns observed following combination therapy. Alterations in the expression of dual targets further complicate the selection of appropriate biomarkers for response assessment. Moreover, most clinical studies to date have not stratified patients based on HR expression intensity or mutational status, leaving the differential efficacy of ADC therapies across distinct HR subgroups poorly defined. Consequently, the formulation of treatment strategies currently lacks a sufficiently refined evidentiary basis.

## 7 Conclusion

HR-positive, HER2-low breast cancer represents a distinct subtype that challenges the traditional binary classification of HER2 status, accounting for nearly 60% of breast cancer cases and emerging as a pivotal focus in precision oncology. This subtype is characterized by a predominance of the ER/PR signaling pathway alongside low-level HER2 cross-signaling, necessitating a therapeutic strategy that integrates endocrine therapy as the foundation with precise selection for anti-HER2 intervention. Diagnostically, a standardized workflow has been established, comprising initial immunohistochemistry (IHC) screening followed by fluorescence in situ hybridization (FISH) confirmation, with the ASCO/CAP guidelines clearly defining the diagnostic gold standard. The combined application of digital pathology, next-generation sequencing (NGS), and liquid biopsy techniques such as circulating tumor DNA (ctDNA) analysis has significantly enhanced diagnostic accuracy and efficiency, while also providing crucial support for therapeutic monitoring and early relapse detection. Regarding treatment, endocrine therapy remains the cornerstone, with CDK4/6 inhibitor combinations serving as the first-line standard for advanced disease; moreover, combination strategies targeting endocrine resistance pathways further improve clinical benefit. Antibody–drug conjugates (ADCs) have reshaped the landscape of anti-HER2 therapy for this population, with trastuzumab deruxtecan emerging as a central option. Several other ADCs, bispecific antibodies, and small-molecule tyrosine kinase inhibitors (TKIs) have also demonstrated considerable potential, although their efficacy awaits validation in large-scale studies. Currently, the management of this subtype has entered a phase of molecularly guided precision, wherein the combination and sequencing of endocrine therapy with ADCs constitute the core therapeutic rationale. Future efforts should focus on the discovery of specific biomarkers to enable refined patient stratification, the exploration of synergistic multi-drug regimens, and the execution of large-sample, multicenter trials to validate novel therapeutics. Ultimately, these advances will underpin the construction of individualized, integrated treatment frameworks aimed at continuously improving patient survival outcomes.
